# Relationship between ENaC Regulators and SARS-CoV-2 Virus Receptor (ACE2) Expression in Cultured Adult Human Fungiform (HBO) Taste Cells

**DOI:** 10.3390/nu14132703

**Published:** 2022-06-29

**Authors:** Mehmet Hakan Ozdener, Sunila Mahavadi, Shobha Mummalaneni, Vijay Lyall

**Affiliations:** 1 Monell Chemical Senses Center, Philadelphia, PA 19104, USA; 2 Department of Biology, Center for Biomedical Research, Tuskegee University, Tuskegee, AL 36088, USA; sunila_mahavadi@yahoo.com; 3 Department of Physiology and Biophysics, Virginia Commonwealth University, Richmond, VA 23298, USA; shobhamummalaneni@comcast.net

**Keywords:** GPER1, RAAS, ACE2, TRPV1, ang II, AT1R, MASR1

## Abstract

In addition to the α, β, and γ subunits of ENaC, human salt-sensing taste receptor cells (TRCs) also express the δ-subunit. At present, it is not clear if the expression and function of the ENaC δ-subunit in human salt-sensing TRCs is also modulated by the ENaC regulatory hormones and intracellular signaling effectors known to modulate salt responses in rodent TRCs. Here, we used molecular techniques to demonstrate that the G-protein-coupled estrogen receptor (GPER1), the transient receptor potential cation channel subfamily V member 1 (TRPV1), and components of the renin-angiotensin-aldosterone system (RAAS) are expressed in δ-ENaC-positive cultured adult human fungiform (HBO) taste cells. Our results suggest that RAAS components function in a complex with ENaC and TRPV1 to modulate salt sensing and thus salt intake in humans. Early, but often prolonged, symptoms of COVID-19 infection are the loss of taste, smell, and chemesthesis. The SARS-CoV-2 spike protein contains two subunits, S1 and S2. S1 contains a receptor-binding domain, which is responsible for recognizing and binding to the ACE2 receptor, a component of RAAS. Our results show that the binding of a mutated S1 protein to ACE2 decreases ACE2 expression in HBO cells. We hypothesize that changes in ACE2 receptor expression can alter the balance between the two major RAAS pathways, ACE_1_/Ang II/AT_1_R and ACE_2_/Ang-(1–7)/MASR1, leading to changes in ENaC expression and responses to NaCl in salt-sensing human fungiform taste cells.

## 1. Introduction

There is considerable evidence that epithelial Na^+^ channels (ENaCs) play a role in human salt taste sensing [1]. In rodents, functional ENaC is composed of α, β, and γ subunits, but some ambiguity remains regarding the exact subunit composition and localization of functional ENaCs in taste receptor cells (TRCs) within the taste buds [2,3]. Unlike rats and mice, humans express an additional ENaC subunit, the δ-subunit. It is likely that in human TRCs, functional ENaC is composed of either αβγ and/or δβγ subunits. ENaC composed of αβγ subunits is more than an order of magnitude less sensitive to amiloride than is ENaC composed of δβγ subunits [4]. Human salt taste is largely but not entirely amiloride-insensitive [1]. Similar to the case in rodents, aldosterone regulates ENaC expression and intracellular trafficking of both α- and δ-ENaC subunits in cultured adult human fungiform (HBO) taste cells [5].

While in rodent models, significant advances have been made in identifying the specific subset of TRCs within the taste buds involved in amiloride-sensitive and amiloride-insensitive salt taste responses, the underlying salt taste transduction mechanisms, and their regulation by hormones, associated receptors, and intracellular signaling intermediates such detailed studies are lacking in human taste cells. Recent studies have identified a novel subset of type II TRCs in mouse fungiform papillae that mediate the amiloride-sensitive salt taste [6,7]. In this study, we first investigated the expression profile of the ENaC δ-subunit in HBO cells. At present, it is not known if δ-ENaC is also expressed in human taste cells that co-express one or more signaling components shown to be co-expressed in the novel subset of salt-sensing type II TRCs in mouse fungiform papillae [6,7].

Our second objective was to investigate if HBO cells expressing the ENaC δ-subunit also co-express receptors of some of the hormones that have been shown to regulate salt responses in rodents. In this regard, preference for salty taste is dependent on reproductive hormones [8]. The G-protein-coupled estrogen receptor (GPER1) is expressed in a subset of mouse type II TRCs that also co-express phospholipase C β2 (PLCβ2) [9]. However, at present, it is not known if GPER1 is expressed in human salt-sensing taste cells that also co-express the ENaC δ-subunit.

Although rodent TRCs do not express transient receptor potential cation channel subfamily V member 1 (TRPV1) [10], TRPV1 mRNA was detected in cultured human taste cell lysates [11]. Accordingly, our third objective was to investigate the presence of functional TRPV1 channels in HBO cells that also co-express δ-ENaC. We further investigated if modulating TRPV1 activity can regulate δ-ENaC expression and function in HBO cells [12].

Renin-angiotensin-aldosterone system (RAAS) components are expressed in salt-sensing mouse TRCs and regulate ENaC expression and behavioral and neural responses to NaCl [13,14,15]. Therefore, our fourth objective was to investigate if one or more RAAS components are also co-expressed in δ-ENaC-positive HBO cells. We hypothesize that if RAAS components are present in HBO cells, δ-ENaC exists in a multi-protein complex with RAAS components TRPV1 and GPER1, and they can modulate one another’s expression and function [16,17]. Accordingly, we investigated the localization of δ-ENaC, GPER1, and TRPV1, along with the angiotensin (Ang) II type 1 receptor (AT1R), angiotensin-converting enzyme 2 (ACE2), and G-protein couple MAS1 oncogene receptor (MASR1) and their interactions with δ-ENaC in HBO cells.

It has been shown that the SARS-CoV-2 virus utilizes a RAAS component, the ACE2 receptor, and the cellular transmembrane serine protease 2 (TMPRSS2) receptor to enter target cells [18]. The SARS-CoV-2 spike protein S1 subunit is responsible for recognizing and binding to the ACE2 receptor [19]. Accordingly, our fifth objective was to investigate if binding of a mutated S1 protein to ACE2 will induce a decrease in ACE2 expression in HBO cells. We hypothesize that changes in ACE2 expression can alter the balance between the two major RAAS pathways (ACE_1_/Ang II/AT_1_R and ACE_2_/Ang-(1–7)/MASR1) leading to changes in ENaC expression and responses to NaCl in salt-sensing human fungiform taste cells [20].

## 2. Material and Methods

### 2.1. Antibodies

The following antibodies were used in these studies: δ-ENaC (Lifespan Biosciences, LS-C119717) or Santa Cruz Biotechnology, goat polyclonal, sc-22246), gustducin (Santa Cruz Biotechnology, sc-395), PLCβ2 (Santa Cruz Biotechnology, sc-515912), ACE_2_ (Abcam ab108252), Taste receptor type 1 member 3 (T1R3; Santa Cruz Biotechnology sc-398996), TRPV1 (Santa Cruz Biotechnology, sc 12,498 or Lifespan Biosciences, LSC172124), GPER1 (Abcam 39742), and AT1R (Millipore: AB15552). The δ-ENaC peptide was obtained from Santa Cruz Biotechnology (sc-22246P).

### 2.2. Chemicals

Fura-2-acetoxymethyl (AM) ester, capsaicin (CAP), iodo-resiniferatoxin (I-RTX, a specific TRPV1 blocker), AVE0991 (a non-peptide MASR1 agonist), Ang II, losartan (an AT1R blocker), dimethyl sulfoxide (DMSO), and amiloride were obtained from Sigma Aldrich. In addition, we used CALHM1 and CALHM3 Taqman primer assay mix (HS0736332_m1 and HS07290139_m1). SARS-CoV-2 (2019-nCoV) spike S1 (D614G)-His recombinant protein was obtained from Sino Biological. TRPV1 and ACE2 small interfering RNA (siRNA) (Qiagen FlexiTube Premix siRNA) were used to downregulate TRPV1 and ACE2, respectively. Scrambled siRNA (Qiagen) was used as a negative control. Pluronic F127 was obtained from Life Technologies.

### 2.3. HBO Cell Culture

HBO cells were derived from two male and two female volunteers and were developed in Dr. Ozdener’s lab at Monell Chemical Senses Center, as a model of human TRCs in vivo. HBO cells stably display all molecular and physiological features characteristic of mature taste cells and exhibit an increase in intracellular calcium ([Ca^2+^]_i_) in response to taste stimuli representing all five taste qualities, indicating the presence of all known signaling pathways [5,21,22,23,24,25]. HBO cells were cultured as described earlier [5,21,22] and were used between passages 4 and 8. HBO cells express all four ENaC subunits [5,23]. Arginine vasopressin, cAMP, and aldosterone regulate ENaC expression and intracellular trafficking of both α- and δ-ENaC in rodent and human TRCs [5]. Arginyl dipeptides, increased NaCl responses in amiloride-sensitive HBO cells [23] and have been shown to enhance salt taste intensity in human subjects [26]. In spite of some ambiguity regarding the role of ENaC in human salt taste perception [1], these studies demonstrate that enhancing ENaC activity in human salt-sensing TRCs correlates with enhanced salt taste intensity in human subjects.

### 2.4. Enrichment of HBO Cells

Cell culture plates (Corning USA) or glass coverslips were coated with δ-ENaC antibody (1–2 mg) or TRPV1 antibody in 100 mL coating buffer solution (0.8 g NaCl, 0.02 g KCl, 0.144 g Na_2_HPO_4_, 0.024 g KH_2_PO_4_ in water to 100 mL, pH 7.4) using a conical cell culture cylinder. Plates were incubated at 36 °C for 2 h. Cells were collected using a cell scraper into the culture medium and centrifuged for 5 min at 2500 rpm/min at room temperature. Cells (~500) were resuspended in fresh medium and plated on the antibody coated surface. After 1–2 h at 36 °C, the medium and unattached cells were gently removed, and fresh medium was added. After enrichment, approximately 70–98% of enriched cells were found immunoreactive to the targeted protein.

### 2.5. siRNA Transfection

Two methods were used for siRNA transfection. In the first method, the RNA interference analysis was performed by transfecting HBO cells with siRNA. Three days before transfection, 2000 cells per well were seeded in 12-well plates. HBO cells were transfected with 25 nmol/L human TRPV1 gene-specific siRNA (SI00058849) or scrambled siRNA. At 3–5 days post-transfection, single cell calcium imaging was performed of siRNA-treated and scrambled siRNA-treated cells, along with un-transfected control cells. The results presented are representative of at least three independent experiments.

In the second method, HBO cells were seeded onto 60 mm dishes. After reaching 70–80% confluence, cells were co-transfected with 1 µg pSIREN DNR DS-RED plasmid and 30 nM scrambled, ACE2 or TRPV1 siRNA using Lipofectamine 2000 reagent. Transfection efficiency was determined using immunofluorescence and was between 75 and 80% (data not shown). Cells were then cultured in media containing high salt (HS; additional 20 mM NaCl) and capsaicin (CAP; 2.5 µM) for 3 or 6 days, and changes in expression of ACE2, TRPV1 and δ-ENaC mRNA were monitored.

### 2.6. Measurement of [Ca^2+^]_i_ in HBO Cells Using a Multimode Microplate Reader

Changes in [Ca^2+^]_i_ in response to stimuli were measured using FlexStation 3. HBO cells (~90,000 cells/plate) were cultured for 24 h in a 96-well plate in media containing HS (20 mM NaCl), CAP (2.5 µM), and Ang II (1 µM) or AVE0991 (0.1 and 1 µM). Following this, cells were washed with normal Ringer’s solution (150 mM NaCl, 5 mM KCl, 1 mM CaCl_2_, 1 mM MgCl_2_, 10 mM glucose and 10 mM HEPES, pH 7.4) and loaded with Fura-2-AM for 1 h at 36 °C in a 5% CO_2_ incubator. The cells were alternately excited at 340 nm and 380 nm, and the emitted light intensity was measured at 530 nm. Temporal changes in fluorescence intensity ratio (FIR; F_340_/F_380_) reflects time-dependent changes in [Ca^2+^]_i_. Cells were washed with zero-Na^+^ Ringer’s solution (150 mM n-methyl-D-glucamine Cl, 5 mM KCl, 1 mM CaCl_2_, 1 mM MgCl_2_, 10 mM glucose and 10 mM HEPES, pH 7.4) and a baseline FIR level was measured for 60 s. Changes in FIR were measured every 4 s at 36 °C. Na^+^ influx was initiated by increasing bath NaCl from 0 to 140 mM. The data were transferred from the SoftMax Pro software to Excel for further analysis.

### 2.7. Single-Cell Ca^2+^ Imaging

Single cell Ca^2+^ imaging was performed as described earlier [23]. Briefly, cultured HBO cells were seeded onto coverslips, grown for 3–5 days, and then loaded with 5 µM fura-2 AM and 10% Pluronic F127 dissolved in DMSO in zero-Na^+^ Ringer’s solution for 1 h at 36 °C. The images were visualized with an inverted fluorescence microscope (Olympus) and a CCD camera (Photometrics). The stimulus delivery and removal were controlled by a two-channel peristaltic pump (Spetec). Cells were exposed to the stimulus for 1 min and provided at least 5 min of recovery time between stimuli. Images were captured every 2 s during stimulus applications, with excitation wavelengths of 340 nm and 380 nm and an emission wavelength centered at 510 nm. Cell focusing, defining regions, and image acquisition were controlled by Metafluor software (Molecular Devices). Both 50 and 150 mM NaCl were examined in the absence and presence of 50 µM amiloride.

The change in FIR (F340/F380) was recorded for regions of interest (ROIs) selected for each cell. Increases in [Ca^2+^]_i_ evoked by stimulus application are expressed as ΔF = FPeak − FBaseline, where F = absorbance at 340/380 nm. The criterion for a responding cell was ΔF ≥ 0.03. Cells that failed to return to baseline were not counted, nor were apparent responses that occurred within 30 s or more than 120 s after stimulation. The percentage of responding cells was calculated by dividing the number of cells with a detectable Ca^2+^ increase by the total number of cells in the given experimental condition. The baseline fluorescence (ΔF/F) of a cell was measured before taste stimuli were given, and the peak value of Ca^2+^ was measured after taste stimuli given. Statistical analysis of Ca^2+^ changes was conducted by counting the Ca^2+^ peaks during the first 2 min of stimulation. Data are presented as a percent of the total number of HBO cells examined in a separate set of experiments that show amiloride-sensitive (AS) NaCl response or amiloride-insensitive (AI) NaCl response or capsaicin (CAP)-induced increase in [Ca^2+^]_i_. Data were processed and plotted using Origin 8 (OriginLab) and Excel (Microsoft). Statistical comparisons between AS, AI, and CAP-sensitive cells were performed using student’s *t* test; *p* values < 0.05 were considered significant [23].

### 2.8. Animals

Since TRPV1 is not expressed in rodent TRCs [10], we used mouse gastric smooth muscle (MGSM) strips from male, female, and ovariectomized (OVX) female mice as controls to test if GPER1 is co-expressed with TRPV1. Approximately 6 weeks-old age- matched male, female, and OVX C57BL/6J mice were purchased from Jackson Laboratories (Bar Harbor, ME). Mice were housed 3–4 per cage in an animal facility directed by the Division of Animal Resources at Virginia Commonwealth University (VCU) with *ad libitum* access to food and water and subjected to a 12/12 h light/dark cycles. All studies were approved by the Institutional Animal Care and Use Committee (IACUC) at VCU prior to the start of any experiments.

### 2.9. Preparation of Gastric and Colonic Smooth Muscle Strips

Mice were anesthetized by CO_2_ inhalation/asphyxiation followed by cervical dislocation. The stomach was removed, and the gastric muscle layer was separated from the mucosa by scraping. Smooth muscle strips from gastric muscle layer were used for Western blot, and co-immunoprecipitation studies.

### 2.10. Isolation of RNA and Quantitative PCR

Total RNA was isolated from cultured HBO cells using an Ambion RNA isolation kit and then treated with TURBO DNase. RNA was reversely transcribed using the High-Capacity cDNA Reverse Transcription kit in a 20-µL reaction volume. Quantitative RT-PCR (qRT-PCR) was performed on cDNA samples using specific primers designed from known sequences in humans using SYBRgreen or Taqman PCR Mastermix. The target gene copy number was quantified by measuring threshold cycle parameter, defined as the fractional cycle at which the fluorescence generated by cleavage of probe passes a fixed threshold above the baseline, and by using a standard curve to determine the starting copy number. The primers are designed to satisfy the requirements for use of the 2^−ΔΔCt^ quantification method and normalize to β-actin expression. Final results are expressed as fold changes in expression in test samples relative to control. All PCR reactions were performed in an ABI stepOne Plus PCR. Specific human primer sequence for MASR1, ACE2, TMPRSS2, TRPV1, GPER1, and β-actin are shown in Table 1. We used Taqman primers for Calhm 1(Id: Hs00736332_m1), Calhm3 (Hs07290139_m1), and glyceraldehyde-3-phosphate dehydrogenase (GAPDH). The amplicons were detected by electrophoresis using 20 µL of the amplified reaction mixture in a 10% agarose gel. A 100-bp (New England Biolabs# N0467S) or 1kb (New England Biolabs# N3232S) molecular weight marker was used to evaluate the PCR product. TRPV1 PCR product was purified and sequenced.

### 2.11. Western Blot Analysis

HBO cells or mouse gastric smooth muscle strips were solubilized in Triton X-100-based lysis buffer plus protease and phosphatase inhibitors. After centrifugation of the lysates at 20,000× *g* for 10 min at 4 °C, protein concentrations of the supernatant were determined with the DC Protein Assay kit from Bio-Rad (Hercules, CA, USA). Equal amounts of proteins were fractionated by SDS-PAGE and transferred to PVDF membranes. Blots were blocked using blocking buffer (BioRad) for 10 min at room temperature and then incubated overnight at 4 °C with various primary antibodies in a blocking buffer. After incubation for 1 h with horseradish peroxidase-conjugated corresponding secondary antibody (1:5000, GE Amersham) in the blocking buffer, immunoreactive proteins were visualized using Clarity Max^TM^ kit (BioRad). All washing steps were performed with TBS-T. A 10–250 kDa PageRuler^TM^ plus pre-stained protein ladder (Fisher Scientific #PI26620) was used to evaluate the protein expression.

### 2.12. Protein-Protein Association

Sequential immunoprecipitation and immunoblot with selective antibodies were used to determine the association of ACE2 with δ-ENAC; T1R3 with TRPV1; T1R3 with ACE2; TRPV1 with ACE2; and GPER1 with AT1R, TRPV1, ACE2, and δ-ENaC. HBO cells or gastric smooth muscle were lysed by incubation for 30 min at 4 °C in Triton X-100-based lysis buffer plus protease and phosphatase inhibitors. After centrifugation of the lysates at 20,000× *g* for 10 min at 4 °C, protein concentrations of the supernatant were determined with the DC Protein Assay kit from Bio-Rad (Hercules, CA, USA). A total of 100 µg of protein was precleared by incubation with 40 μL of protein A/G agarose for 4 h and then incubated overnight with antibody to ACE2, T1R3, TRPV1, or GPER1. Protein A/G agarose was then added and incubated for another 2 h, and the mixture was centrifuged at 13,000× *g* for 5 min. The immune-precipitates were washed four times in lysis buffer and boiled in Laemmli buffer. Samples were separated by SDS-PAGE, transferred to PVDF membranes, and probed with antibody to δ-ENAC, T1R3, TRPV1, ACE2, and AT1R. After incubation with secondary antibody, the proteins were visualized using Clarity Max^TM^ kit (BioRad). All washing steps were performed with TBS-T. A 10–250 kDa PageRuler^TM^ plus pre-stained protein ladder (Fisher Scientific #PI26620) was used to evaluate the protein expression.

## 3. Results

### 3.1. Localization of ENaC in HBO Cells

**Figure 1:**
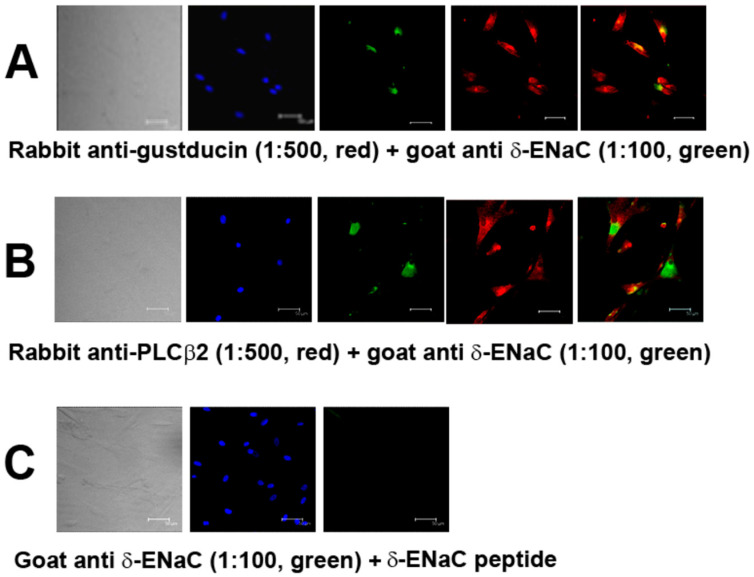
δ-ENaC positive HBO cells: (**A**,**B**) co-localization of δ-ENaC in gustducin-positive (**A**); and PLCβ2-positive (**B**) HBO cells; (**C**) peptide inhibition of δ-ENaC antibody binding to HBO cells. Left panels transmitted images: blue 4′,6-diamidino-2-phenylindole (DAPI) stained cell nuclei; green δ-ENaC antibody binding; red gustducin or PLCβ2 antibody binding. Scale bar = 50 µm.

We have previously shown that all four ENaC subunits (α, β, γ, and δ) are expressed in a subset of HBO cells [5,23]. Here, we show that the δ-ENaC antibody co-localizes with gustducin (Figure 1A) and PLCβ2 (Figure 1B) antibodies in HBO cells. Treating the cells with δ-ENaC antibody plus goat anti-δ-ENaC peptide completely abolished δ-ENaC immunofluorescence (Figure 1C). Both α-ENaC (Figure S1A) and γ-ENaC (Figure S1B) subunit antibodies also co-localized with PLCβ2 antibodies in HBO cells. These results suggest that α, γ, and δ-ENaC antibodies bind specifically to a subset of type II human fungiform taste cells.

### 3.2. Localization of RAAS Components in HBO Cells

**Figure 2:**
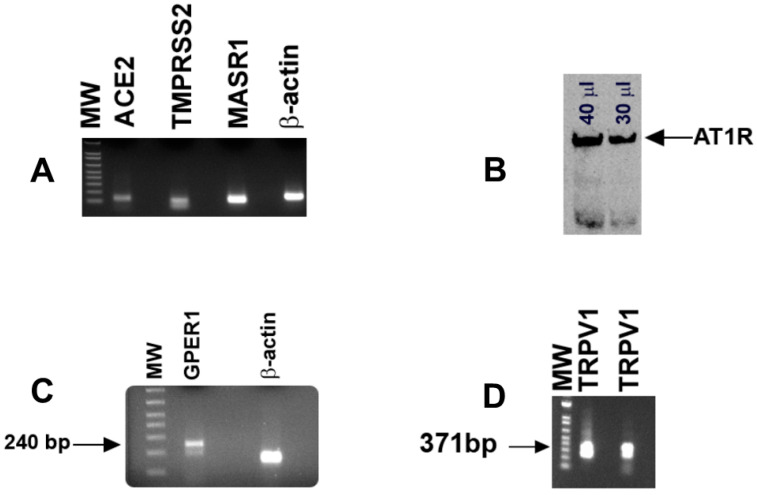
Detection of ACE2, TMPRSS2, MASR1, β-actin, AT1R, GPER1, and TRPV1 in HBO cells: (**A**) in HBO cell lysates, RT-PCR primers (Table 1) for ACE2 (124 bp), MASR (117 bp), TMPRSS2 (105 bp), and β-actin (153 bp) yielded single bands of predicted sizes; (**B**) in Western blot experiments, AT1R antibody (1:500 dilution) detected a single band of AT1R in HBO cell lysate. Antibody binding was responsive to different concentrations of protein loading of the HBO cell lysate (30 or 40 µL); (**C**,**D**) using human primers for GPER1 and TRPV1 (Table 1), RT-PCR yielded a single band of 240 bp (**C**) and 371 bp (**D**), respectively. TRPV1 mRNA RT-PCR product was sequenced and was found to be specific for the TRPV1 gene NM_080705.4 (Figure S2). Molecular weight (MW) DNA ladder bands correspond to 100–800 bp (bottom to top).

In HBO cell lysates, RT-PCR primers (Table 1) for ACE2 (124 bp), MASR1 (117 bp), and TMPRSS2 (105 bp) yielded single bands of predicted sizes (Figure 2A). RT-PCR primers (Table 1) for β-actin were used as a control and yielded a single band of 153 bp (Figure 2A). In Western blot experiments, AT1R antibody detected a single band of AT1R in HBO cell lysate (Figure 2B). These results suggest that in addition to RAAS components, HBO cells express TMPRSS2.

### 3.3. Localization of GPER1 and TRPV1 mRNA in HBO Cells

Using human primers for GPER1 (Table 1), RT-PCR in HBO cell lysates yielded a single band of 240 bp (Figure 2C). These results suggest that GPER1 is expressed in HBO cells. RT-PCR primers (Table 1) for TRPV1 [27] yielded a single band of predicted size (371 bp; Figure 2D). TRPV1 mRNA RT-PCR product was sequenced and was found to be specific for the TRPV1 gene NM_080705.4 (Figure S2).

### 3.4. Localization of CALHM1/3 mRNA in HBO Cells

**Figure 3:**
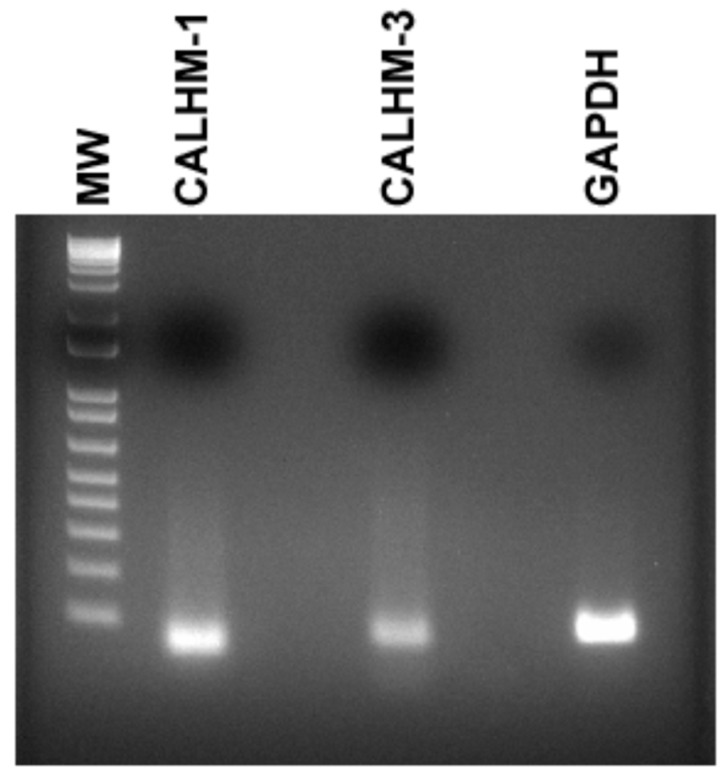
Detection of CALHM-1 and CALHM-3 in HBO cells. In HBO cell lysates, using CALHM1 and CALHM3 Taqman primer assay mix, RT-PCR yielded single bands of 55 bp and 64 bp, respectively. GAPDH was used as an endogenous control. Molecular weight (MW) DNA ladder bands correspond to 100–1 Kb (bottom to top).

In mouse fungiform papillae and soft palate, Na^+^-specific salt taste was detected by a subset of type II cells that express PLCβ2, inositol 1,4,5-trisphosphate receptor type 3 (ITPR3), voltage-dependent ATP release channel composed of calcium homeostasis modulator 1 (CALHM1) and 3 (CALHM3), and skinhead-1a (SKN-1a, a transcription factor) [6,7]. Accordingly, we further tested if CALHM1/3 are expressed in HBO cells. In HBO cell lysates, using CALHM1 and CALHM3 Taqman primer assay mix, RT-PCR yielded single bands of 55 bp and 64 bp, respectively (Figure 3). These results suggest that CALHM1 and CALHM3 are expressed in HBO cells.

### 3.5. Interaction of ENaC Regulatory Proteins in HBO Cells

**Figure 4:**
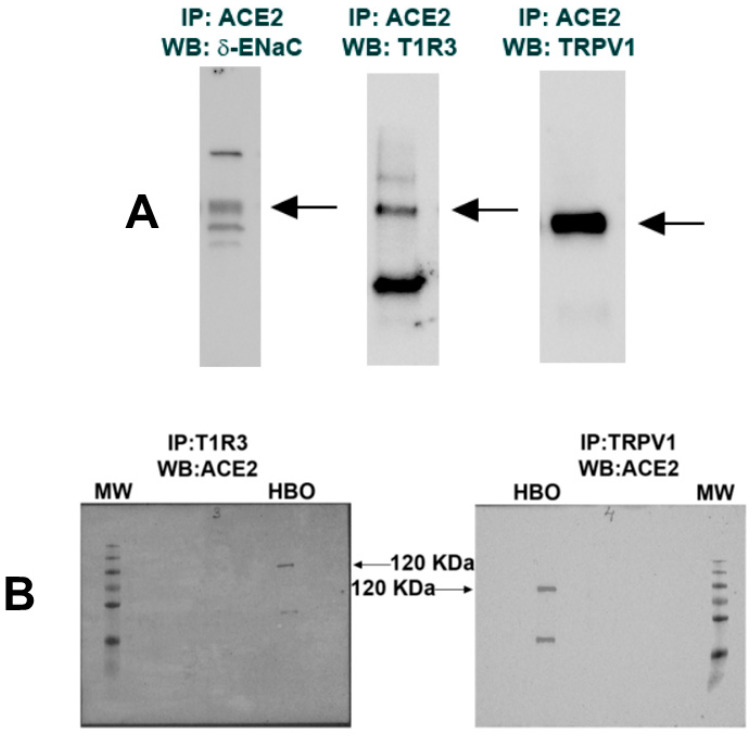
Interactions among ACE2, δ-ENaC, T1R3, and TRPV1: (**A**) co-immuno-precipitation of ACE2/δ-ENaC, ACE2/T1R3, and ACE2/TRPV1; (**B**) co-immuno-precipitation of T1R3/ACE2 and TRPV1/ACE2. Molecular weight (MW) protein ladder bands correspond to 10–250 KDa (bottom to top).

Co-IP studies were performed using ACE2, δ-ENaC, T1R3, and TRPV1 antibodies and Western blots to investigate if they physically interact with each other [22]. Results shown in Figure 4A demonstrate that δ-ENaC, T1R3, and TRPV1 interact specifically with the immobilized ACE2 in HBO cell lysates. In turn, ACE2 antibody pulled down T1R3 and TRPV1 (Figure 4B). In additional experiments, TRPV1 antibody pulled down δ-ENaC. When IgG was used instead of TRPV1 antibody, no δ-ENaC co-immunoprecipitation was observed (data not shown). Taken together, these results suggest that ACE2 exists in a complex with δ-ENaC and TRPV1 in salt-sensing HBO cells and in a complex with T1R3 in sweet-sensing HBO cells [15].

Since TRPV1 is not expressed in rodent TRCs [10], we used mouse gastric smooth muscle (MGSM) from male, female, and OVX female mice as controls to test if GPER1 is co-expressed with TRPV1. In Western blots (Figure S3) relative to MGSM from female mice, GPER1 protein expression was lower in males and further decreased in OVX females and validates the use of GPER1 antibody. GPER1 protein was also expressed in HBO cells. In Co-IP studies, GPER1 antibody pulled down AT1R [28], TRPV1, ACE2, and δ-ENaC in HBO cell lysate (Figure S4). GPER1 was also associated with AT1R, TRPV1, and ACE2 in MGSM. These results suggest that RAAS components GPER1 and the ENaC δ-subunit are present in a complex with TRPV1 in HBO cells.

### 3.6. Immuno co-Localization of TRPV1 and δ-ENaC

**Figure 5:**
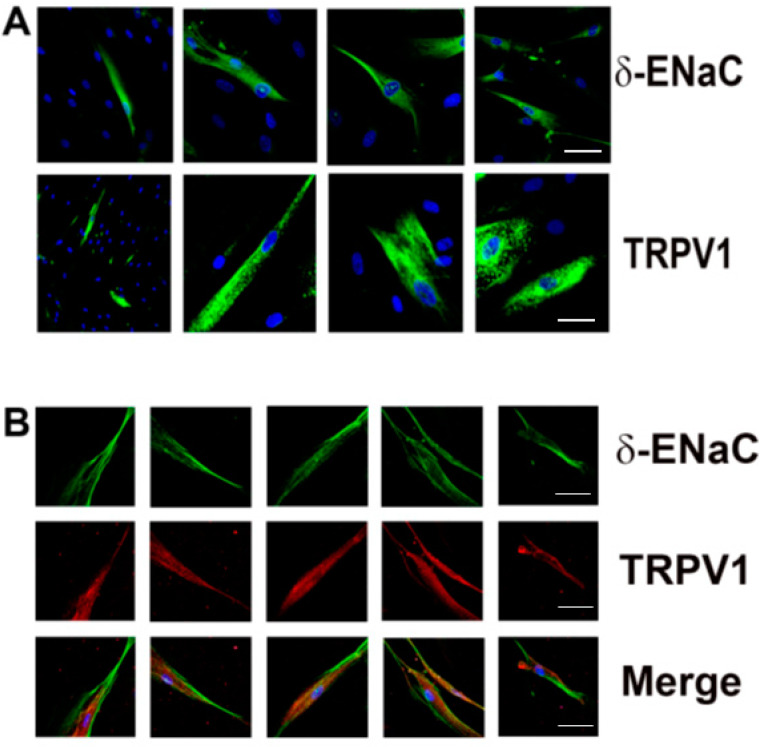
Co-localization of δ-ENaC and TRPV: (**A**) localization of δ-ENaC and TRPV1 in HBO cells; blue DAPI-stained cell nuclei and green δ-ENaC or TRPV1 antibody binding; the left column shows low magnification (20×) images; the remaining panels are high-magnification images (40×) that show δ-ENaC or TRPV1 antibody binding in individual cells; (**B**) localization of TRPV1 in individual δ-ENaC-positive HBO cells at 40× magnification; green δ-ENaC antibody binding; red TRPV1 antibody binding. Scale bars 10 µm.

At low magnification (20×), δ-ENaC antibody binding (Figure 5A; upper left panel) and TRPV1 antibody binding (Figure 5A; lower left panel) were observed only in a subset of HBO cells. At high magnification (40×; Figure 5A; right three panels), δ-ENaC- and TRPV1-positive individual cells could be easily visualized. Dual immunofluorescence studies show that TRPV1 co-localizes in δ-ENaC positive HBO cells (Figure 5B, “Merge”). No significant labelling was observed when the primary antibody step was omitted (data not shown).

### 3.7. Modulation of ACE2 mRNA Expression in HBO Cells by High Salt (HS), Mutated S1 Protein, AVE0991, and Losartan

**Figure 6:**
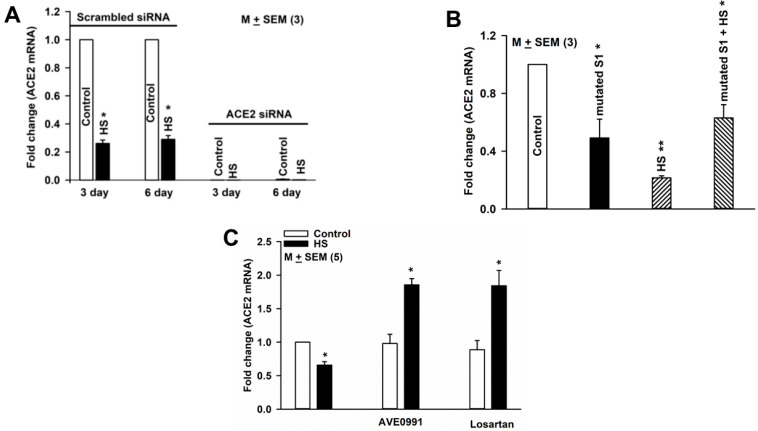
Regulation of ACE2 mRNA expression in HBO cells: (**A**) effect of HS (20 mM NaCl) and ACE2 siRNA on ACE2 mRNA expression in HBO cells, * *p* = 0.0001; (**B**) effect of HS and mutated S1 protein on ACE2 mRNA expression in HBO cells; * *p* = 0.0172 (S1); ** *p* = 0.0001 (HS); and * *p* = 0.0114 (HS + S1); (**C**) effect of AVE0991 and losartan on ACE2 mRNA expression in HBO cells. HBO cells were cultured in control media and control media + HS in the absence and presence of AVE0991 (1 µM) or losartan (1 µM) for 3 days. Values are mean of qPCRs performed in two separate experiments. * *p* = 0.002 (HS); * *p* = 0.0008 (AVE0991); and * *p* = 0.007(losartan).

Following transfection with either 30 nM scrambled siRNA or 30 nM ACE2 siRNA, cells were cultured in media containing HS (20 mM NaCl) for 3 or 6 days and changes in ACE2 mRNA expression were monitored. In HBO cells transfected with scrambled siRNA, HS treatment for 3 and 6 days inhibited ACE2 mRNA expression by 74 and 71%, respectively, (Figure 6A). Transfection with ACE2 siRNA almost completely abolished ACE2 mRNA expression in control and HS media (Figure 6A). These results suggest that culturing HBO cells in the presence of media containing HS inhibits ACE2 mRNA expression.

To investigate the effect of SARS-CoV-2 S1 protein on ACE2 mRNA expression in HBO cells, we used SARS-CoV-2 (2019-nCoV) spike S1 (D614G)-His recombinant protein from Sino Biological. HBO cells were cultured in control media and in media containing HS (20 mM NaCl) for 3 days in the absence and presence of 600 ng/mL of the mutated S1 protein. The binding EC_50_ of the spike S1 protein to immobilized human ACE2 protein has been reported to be between 200 and 600 ng/mL (Sino Biological). Mutated S1 protein and HS decreased ACE2 expression by 51% and 78.5%, respectively, (Figure 6B). In the presence of mutated S1 protein, no additional HS-induced decrease in ACE2 mRNA expression was observed. These results suggest that the binding of mutated S1 protein to ACE2 decreases ACE2 mRNA expression and prevents a further decrease in ACE2 mRNA expression in the presence of HS.

We further tested if enhancing Ang-(1–7) or inhibiting AT1R will overcome the HS-induced loss of ACE2 function in HBO cells [29]. HBO cells were cultured in control media and control media + HS in the absence and presence of AVE0991 (1 µM; a MASR1 agonist), and losartan (1 µM; an AT1R blocker) for 3 days. Relative to control media, HBO cells cultured in HS media showed a decrease in ACE2 mRNA expression (Figure 6C). AVE0991 and losartan did not alter ACE2 expression in control media. However, in cells cultured in control media. + HS showed significant enhancement in ACE2 mRNA expression. These results suggest that increasing Ang-(1–7) or inhibiting AT1R can reverse the effects of HS on ACE2 mRNA expression.

### 3.8. Effect of HS on ENaC and TRPV1 Expression in HBO Cells

**Figure 7:**
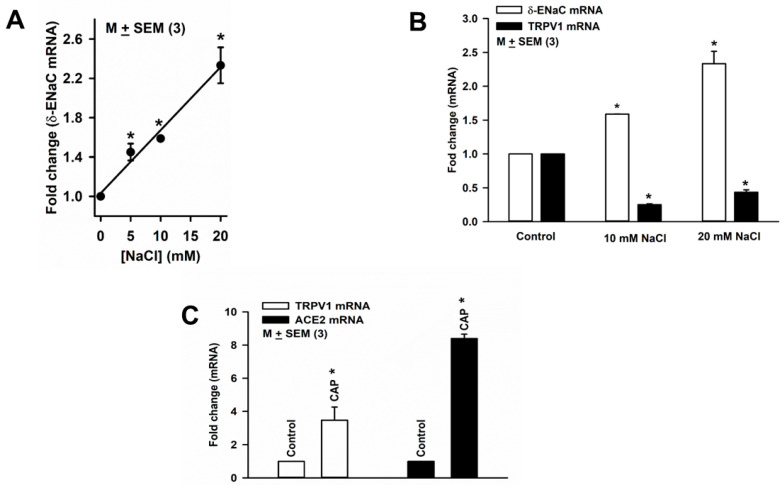
Regulation of δ-ENaC mRNA expression in HBO cells: (**A**) effect of high salt (HS) on δ-ENaC mRNA expression in HBO cells, * *p* = 0.0001; (**B**) HS-induced changes in δ-ENaC and TRPV1 mRNA expression in HBO cells; * *p* = 0.0001 (10 mM NaCl); * *p* = 0.0019 (20 mM NaCl); * *p* = 0.0001 (TRPV1 mRNA); (**C**) CAP-induced changes in TRPV1 and ACE2 mRNA expression in HBO cells. * *p* = 0.0356 (TRPV1 mRNA); and * *p* = 0.0001 (ACE2 mRNA).

Culturing HBO cells in media containing an additional 5, 10, or 20 mM NaCl for 3 days produced a concentration-dependent increase in δ-ENaC mRNA expression in HBO cells (Figure 7A). Culturing HBO cells in media containing an additional 10 or 20 mM NaCl for 3 days increased δ-ENaC mRNA expression and also decreased TRPV1 mRNA expression (Figure 7B). These results raise the possibility that HS-induced changes in TRPV1 and δ-ENaC are related. In contrast to HS, culturing HBO cells with 2.5 µM CAP increased the expression of both TRPV1 and ACE2 mRNA (Figure 7C). These results suggest that HS and CAP produce opposite effects on TRPV1 and ACE2 mRNA expression in HBO cells.

### 3.9. Effect of TRPV1 siRNA and ACE2 siRNA on TMPRSS2 mRNA and MASR1 mRNA Expression in HBO Cells

**Figure 8:**
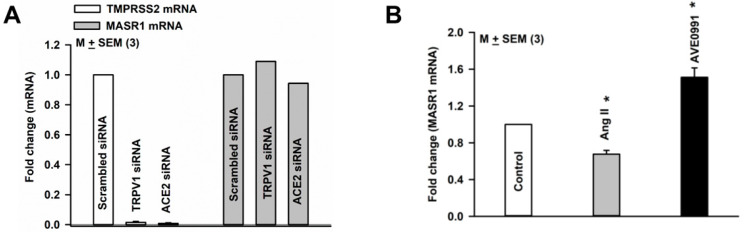
Regulation of MASR1 and TMPRSS2 mRNA in HBO cells: (**A**) TRPV1 siRNA and ACE2 siRNA-induced changes in MASR1 and TMPRSS2 mRNA expression in HBO cells; (**B**) effect of Ang II and AVE0991 on MASR1 mRNA expression in HBO cells. Values are means of qPCR performed in triplicate. * *p* = 0.0014 (Ang II); * *p* = 0.0075 (AVE0991).

TMPRSS2 mRNA expression was inhibited when HBO cells were transfected with TRPV1 or ACE2 siRNA (Figure 8A). In contrast, MASR1 mRNA expression was unaffected when HBO cells were transfected with TRPV1 or ACE2 siRNAs (Figure 8A). These results suggest that in HBO cells, the expression of TMPRSS2 is modulated by altering the expression of TRPV1 and/or ACE2.

### 3.10. Effect of Ang II and AVE0991on MASR1 mRNA Expression in HBO Cells

Culturing HBO cells in control media containing Ang II (1 µM) for 3 days decreased MASR1 mRNA expression (Figure 8B), and in culture media containing AVE0991 (1 µM), a non-peptide agonist of MASR1 [30], increased MASR1 mRNA expression (Figure 8B).

### 3.11. Changes in NaCl-Responsive HBO Cells following δ-ENaC and TRPV1 Enrichment

**Figure 9:**
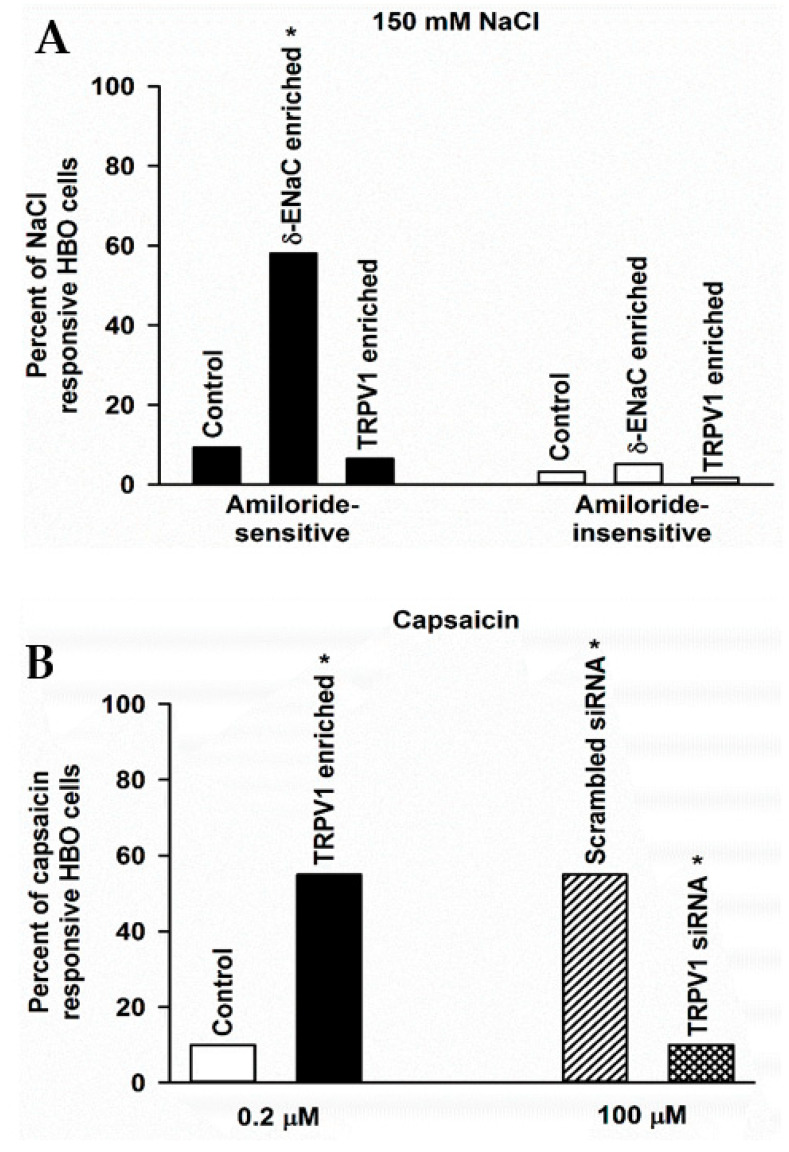
Effect of δ-ENaC enrichment, TRPV1 enrichment, and TRPV1 siRNA on number of NaCl and capsaicin (CAP) responsive HBO cells: (**A**) amiloride-sensitive (AS) and amiloride-insensitive (AI) responses to 150 mM NaCl in single HBO cells using calcium imaging. In 11 independent experiments under control conditions, out of 600 HBO cells investigated, 9.3% of cells demonstrated AS NaCl response, while 3.2% of cells demonstrated AI NaCl response. In 6 separate experiments following δ-ENaC enrichment, out of 286 HBO cells investigated, 166 cells (58%) demonstrated AS NaCl response. While in 5 separate experiments, out of 134 HBO cells examined, 7 cells (5%) demonstrated AI NaCl response. Thus, δ-ENaC enrichment produced a 6-fold (* *p* < 0.001) increase in AS-NaCl response in HBO cells. Following TRPV1 enrichment, in 3 separate experiments, out of 180 HBO cells examined, 6.5% of the cells demonstrated AS NaCl response, while 1.7% of the cells demonstrated AI NaCl response. (**B**) Changes in [Ca^2+^]_i_, in single HBO cells using calcium imaging in HBO cells treated with CAP, scrambled siRNA, or TRPV1 siRNA. Following treatment with 0.2 µM CAP, 10% of control HBO cells and 55% of TRPV1-enriched cells responded with an increase in [Ca^2+^]_i_. Thus, TRPV1 enrichment produced a 5.5-fold (* *p* < 0.001) increase in CAP-responsive HBO cells. Following treatment with 100 µM CAP, out of 148 un-transfected HBO cells examined, 82 cells (55%) demonstrated an increase in [Ca^2+^]_i_. In contrast, out of 242 TRPV1 siRNA treated HBO cells investigated, 24 cells (10%) demonstrated a CAP-induced increase in [Ca^2+^]_i_. Thus, TRPV1 siRNA produced a 5.5-fold (* *p* < 0.001) decrease in CAP-responsive HBO cells.

We measured changes in amiloride-sensitive (AS) and amiloride-insensitive (AI) responses to 150 mM NaCl in single HBO cells using calcium imaging. In three independent experiments, 9.3% of cells demonstrated AS, and 3.2% demonstrated AI NaCl responses (Figure 9A). Following δ-ENaC enrichment, 58% of HBO cells demonstrated AS, and 5.2% demonstrated AI NaCl response (Figure 9A). These results indicate that enriching HBO cells with δ-ENaC specifically increases AS NaCl response in salt-sensing human taste cells. In TRPV1 enriched HBO cells, 6.5% of cells demonstrated AS, and 1.7% demonstrated AI NaCl response (Figure 9A). These results suggest that enriching HBO cells with TRPV1 tends to decrease AS NaCl responses in salt-sensing human taste cells. In contrast, δ-ENaC and TRPV1 enrichment produced only small changes in the number of HBO cells that demonstrated AI NaCl responses. After enrichment, approximately 70–98% of enriched cells were immuno-reactive to targeted protein (data not shown).

### 3.12. Changes in Capsaicin (CAP) Responsive HBO Cells following TRPV1 Enrichment and TRPV1 siRNA Treatment

We measured changes in [Ca^2+^]_i_ in single HBO cells using calcium imaging in HBO cells treated with CAP. In control HBO cells treated with 0.2 µM CAP, only 10% of the cells responded with an increase in [Ca^2+^]_i_ (Figure 9B). In contrast, in TRPV1-enriched HBO cells treated with 0.2 µM CAP, 55% of the cells responded with an increase in [Ca^2+^]_i_ (Figure 9B). These results show that TRPV1 enrichment produced a 5.5-fold increase in CAP-responsive HBO cells.

We also measured CAP-induced changes in [Ca^2+^]_i_ in single HBO cells following transfection of cells with scrambled siRNA or TRPV1 siRNA. In these experiments, CAP was used at 100 µM. In HBO cells treated with scrambled siRNA, 55% of the cells demonstrated CAP-induced increase in [Ca^2+^]_i_ (Figure 9B). These results show that increasing CAP concentration from 0.2 to 100 µM increases CAP-responsive HBO cells by 5.5-fold. In contrast, in TRPV1 siRNA transfected HBO cells, only 10% of the cells demonstrated a CAP-induced increase in [Ca^2+^]_i_ (Figure 9B). These, results show that TRPV1 siRNA produced a 5.5-fold decrease in CAP-responsive HBO cells. These results further show the presence of functional TRPV1 channels in HBO cells.

### 3.13. Changes in [Ca^2+^]_i_ in HBO Cells in Response to Stimuli

**Figure 10:**
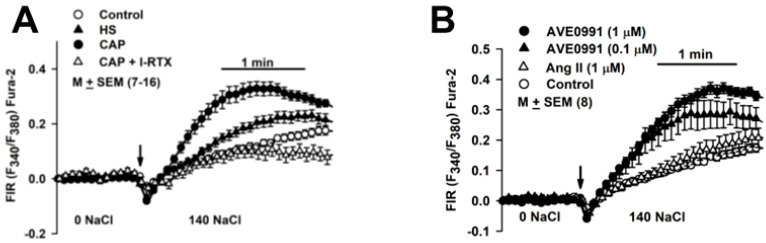
Regulation of Na^+^ influx by HS, CAP, AVE0991, and Ang II. (**A**) Effect of culturing HBO cells in media containing HS and CAP on Na^+^ influx. Na^+^ flux-induced secondary changes in [Ca^2+^]_i_ were monitored in HBO cells in response to an increase in bath Na^+^ from 0 to 140 mM. The increase in initial slope (ΔFIR/min) and V_max_ were measured under each experimental condition. Values are mean (M) ± SEM of changes in FIR in 7–16 wells. (**B**) Effect of culturing HBO cells in media containing AVE0991 and Ang II on Na^+^ influx. Values are mean (M) ± SEM of changes in FIR in 8 wells.

In HBO cells cultured in a control media, increasing Na^+^ from 0 to 140 mM induced a secondary increase in [Ca^2+^]_i_ influx with an initial slope (ΔFIR/min) and V_max_ of 0.099 ± 0.008 and 0.13 ± 0.013, respectively, (Figure 10A; ○). In HBO cells cultured in control media + CAP (2.5 µM), ΔFIR/min and V_max_ increased to 0.397 ± 0.037 and 0.327 ± 0.025, respectively, (*p* = 0.0001) (Figure 10A; ●). In HBO cells cultured in control media + CAP, initiating Na^+^ influx with 140 mM NaCl + 1 μM I-RTX (1 µM) decreased ΔFIR/min and V_max_ to 0.137 ± 0.019 (*p* = 0001) and 0.094 ± 0.028 (*p* = 0001), respectively, (Figure 10A; Δ). I-RTX- sensitive Na^+^ influx reflects the CAP-induced increase in TRPV1 expression in HBO cells. Relative to control, HS alone increased ΔFIR/min and V_max_ to 0.197 ± 0.018 (*p* = 0.0011) and 0.21 ± 0.021 (*p* = 0.015), respectively, (Figure 10A; ▲). The Na^+^ flux-induced secondary changes in [Ca^2+^]_i_ in the presence of HS are most likely related to HS-induced upregulation in ENaC expression (Figure 7A,B). These results suggest that the activation of TRPV1 by CAP affected ENaC expression and that this change was lost when TRPV1 was inhibited.

We next monitored the effect of RAAS modulators on Na^+^ flux-induced secondary changes in [Ca^2+^]_i_ in HBO cells [23]. HBO cells cultured in control media + AVE0991 (0.01 and 1 µM) demonstrated a dose-dependent secondary increase in [Ca^2+^]_i_ influx, whereas Ang II did not produce a significant change in [Ca^2+^]_i_ influx (Figure 10B).

## 4. Discussion

Our results show that α, γ, and δ ENaC subunits are co-expressed in a subset of PLCβ2 positive HBO cells. In addition, δ-ENaC was also co-expressed in a subset of gustducin positive HBO cells (Figure 1 and Figure S1). These results suggest that α, γ, and δ ENaC subunits are expressed in a subset of type II human fungiform taste cells. Consistent with our results, in mouse fungiform papillae, the ENaC α-subunit was expressed in a subset of type II TRCs that co-express PLCβ2, ITPR3, CALHM1/3, and SKN-1a [6,7]. This subset of type II TRCs did not express TRPM5 (transient receptor potential cation channel subfamily M member 5) and GNAT3 (guanine nucleotide-binding protein G(t) subunit alpha-3; gustducin). Both SKN-1a and CALHM3-deficient mice demonstrated markedly decreased amiloride-sensitive NaCl chorda tympani (CT) taste nerve responses [6]. Genetic elimination of α-ENaC in CALHM1-expressing cells, as well as global CALHM3 deletion abolished amiloride-sensitive NaCl CT taste nerve responses and attenuated behavioral attraction to NaCl [7]. Genetically engineered mice lacking α-ENaC in TRCs exhibit a complete loss of salt attraction and sodium taste responses [31].

ENaCs composed of both αβγ and δβγ subunits contribute to Na+ flux in HBO cells. Treating HBO cells with α-ENaC and δ-ENaC siRNAs downregulated α-ENaC and δ-ENaC subunit mRNAs, decreased the number of cells expressing α-ENaC and δ-ENaC protein, and decreased the numbers of cells that responded to 150 mM NaCl alone from baseline, respectively [23]. At the concentration of amiloride (50 µM) used in our experiments, ENaCs composed of both αβγ and δβγ subunits will be blocked [4]. Further studies are needed to determine if, in addition to the δ-ENaC subunit, other ENaC subunits in human salt-sensing taste cells also co-express one or more of the above signaling effectors. A high concentration of amiloride can block other transporters and hybrid non-selective cation channels. We have previously shown that Ala-Arg-induced an increase in ENaC activity in HBO cells that was blocked by amiloride. The effects of Ala-Arg and amiloride were independent of Na+-H+ exchanger 1, Lysophosphatidic acid receptor 1, calcium-sensing receptor, and PLCβ2 or signaling pathways depending upon these proteins [23].

In contrast to the above studies, mice carrying modified alleles that allow the synthesis of green and red fluorescent proteins in cells expressing α- and β-ENaC subunits, demonstrated that α-ENaC was exclusively expressed in type III cells in the fungiform papillae but not in type I and type II cells, whereas β-ENaC was expressed in type I cells with no expression in type III cells. This suggests that α- and β-ENaC subunits are segregated in mouse fungiform papillae [2]. These results further suggest that the amiloride-sensitive recognition of Na^+^-specific salt taste in mice is unlikely to depend on the classical ENaC channel composed of α-, β-, and γ-subunits [2,3]. Thus, at present, there is a lack of consensus regarding the exact identity of the TRCs involved in amiloride-sensitive salt responses.

Our results show that some of the ENaC regulatory hormones and signaling effectors: TRPV1, RAAS components (ACE2, MASR1, AT1R), GPER1 (Figure 2 and Figure S2), and CALHM1/3 (Figure 3) are expressed in HBO cells. Functional TRPV1 channels are expressed in HBO cells (Figure 2D, Figure 9B and Figure 10A). Human TRCs differ from rodent TRCs with respect to TRPV1 expression [10,11]. Most importantly, our data show that TRPV1 is co-localized in a subset of HBO cells that also co-express the ENaC δ-subunit (Figure 5). This raises the possibility that TRPV1 may have a prospective role in regulating ENaC subunit expression and function in HBO cells.

Culturing HBO cells in media containing HS induces an increase in δ-ENaC mRNA (Figure 7A,B) and protein (unpublished data) expression in HBO cells. Consistent with these results, in rats, changes in dietary NaCl alter amiloride-sensitive NaCl CT taste nerve responses. A diet containing 3% NaCl (HS) induced a greater amiloride-sensitive NaCl CT taste nerve response than did a 1% NaCl diet, whereas reducing dietary NaCl from 1% to 0.1% led to a drastic decrease in the amiloride-sensitivity of NaCl CT taste nerve responses [32]. Na^+^-deficient rats licked significantly more during the first NaCl intake bout than did control rats [33]. In contrast to rats, mice do not seem to respond to changes in ENaC subunit expression when fed a Na^+^-deficient diet or a HS diet [34].

Culturing HBO cells in media containing additional HS increased δ-ENaC mRNA expression and decreased TRPV1 mRNA expression (Figure 7B). Alternately, culturing HBO cells with 2.5 µM CAP increased the expression of TRPV1 (Figure 7C). Similar to our results, α-ENaC and TRPV1 were co-localized in M1-cortical collecting duct (CCD) cells [12]. Culturing M1-CCD cells with HS reduced TRPV1 but increased α-ENaC expression in M1-CCD cells. CAP upregulated TRPV1 and reduced α-ENaC expressions in M1-CCD cells, which was inhibited by the TRPV1-specific blocker, I-RTX. In CCD cells, an HS-induced increase in α-ENaC was accompanied by an increase in with-no-lysine kinase (WNK1) and serum and glucocorticoid-inducible protein kinase 1 (SGK1) [12]. At present, it is not known, if these downstream kinases are involved in TRPV1-dependent regulation of δ-ENaC expression in HBO cells. In this regard, the effect of CAP in mitigating HS-induced changes in ENaC expression in human salt-sensing taste cells may be relevant in reducing salt intake in humans [35,36]. In contrast to HBO cells, TRPV1 agonists and antagonists seem to affect only the AI component of the NaCl CT response in rats and mice [37,38,39].

Our results (Figure 4, Figure 6A and Figure 7C) suggest that ACE2 exists in a complex with δ-ENaC and TRPV1 in salt-sensing HBO cells and in a complex with T1R3 in sweet-sensing HBO cells [15]. Our results further suggest that TRPV1 and δ-ENaC exist in a complex in salt-sensing HBO cells (data not shown). Consistent with our results, human ACE2 has been shown to interact with AT1R, AT2R, and MASR1 in adult lung tissue. Ligand binding to AT1R resulted in the downregulation of ACE2 cell-surface expression, while ligand binding to AT2R, but not to MASR1, resulted in upregulation of ACE2 cell-surface expression [16]. In human proximal tubule epithelial cells, AT2R and MASR1 have been shown to co-localize [17]. In our studies, HS decreased the expression of ACE2 (Figure 6A) and TRPV1 (Figure 7B) mRNA, and CAP increased both ACE2 and TRPV1 mRNA expression in HBO cells (Figure 7C). These results suggest that HS-induced regulation of δ-ENaC mRNA expression may involve changes in TRPV1 and ACE2 mRNA. AVE0991 (a MASR1 agonist) and losartan (an AT1R blocker) did not alter ACE2 expression in the control media but significantly enhanced ACE2 mRNA expression in cells cultured in HS media. We hypothesize that increasing Ang-(1–7) or inhibiting AT1R can reverse the effects of HS on ACE2 mRNA expression.

Ang II decreased and AVE0991 (a non-peptide agonist of MASR1) [30] increased MASR1 mRNA expression (Figure 8B). AVE0991 induced a Na^+^-dependent secondary increase in [Ca^2+^]_i_ influx, whereas Ang II did not produce a significant effect in [Ca^2+^]_i_ influx in HBO cells (Figure 10B). It is likely that the effects of AVE0991 and Ang II are mediated via MASR1. It is suggested that long-term effects of Ang II may involve a decrease, whereas the long-term effects of Ang-(1–7) may involve an increase in ENaC expression and activity. Under conditions where ACE_2_ activity is inhibited, Ang II levels may remain elevated over time.

While MASR1 mRNA expression was unaffected, TMPRSS2 mRNA expression was inhibited when HBO cells were transfected with TRPV1 or ACE2 siRNAs (Figure 8A). These results suggest that in HBO cells, the expression of TMPRSS2 mRNA is TRPV1 and/or ACE2-dependent. SARS-CoV-2 spike S1 protein and HS decreased ACE2 expression (Figure 6B). In the presence of a mutated S1 protein, no additional HS-induced decrease in ACE2 mRNA expression was observed. However, at present the significance of these findings in relation to SARS-CoV-2 infection and changes in salt taste in humans is not clear [40].

In summary, in this study, we have localized ENaC subunits in a subset of type II human taste cells. We have demonstrated the expression of the ENaC regulatory hormones and signaling effectors: TRPV1, ACE2, MASR1, AT1R, GPER1, and CALHM1/3 in human taste cells and have provided evidence that functional TRPV1 channels are expressed in human taste cells. An important finding is that TRPV1 is co-localized in human taste cells that express the ENaC δ-subunit. Modulating TRPV1 activity by HS and CAP can alter ENaC mRNA expression. Our results suggest that in human salt-sensing taste cells, some of the ENaC regulators are most likely present in a complex and that changes in the expression of one or more regulators can alter the expression of other effectors. Another important finding is that mutated S1 protein binds to ACE2 and decreases its expression. In the presence of a mutated S1 protein, no additional HS-induced decrease in ACE2 mRNA expression was observed. The expression of TMPRSS2 mRNA is TRPV1 and/or ACE2-dependent. We further show that it is likely that the effects of AVE0991 and Ang II on Na^+^ influx in human taste cells are mediated via MASR1. We hypothesize that changes in ACE2 expression in human fungiform taste cells can alter the balance between the two major RAAS pathways, ACE_1_/Ang II/AT_1_R and ACE_2_/Ang-(1–7)/MASR, leading to changes in ENaC expression and responses to NaCl.

## Figures and Tables

**Table 1:** RT-PCR primers.

Hs_MASR1	F: 5′ CCCAAGTACCAGTCGGCATT 3′
	R: 5′ GTCATTCCGAGAGTGACTCTCTTCT 3′
Hs_ACE_2_	F: 5′ GGGATCAGAGATCGGAAGAAGAAA 3′
	R: 5′ AGGAGGTCTGAACATCATCAGTG 3′
Hs_TMPRSS2	F: 5′ AATCGGTGTGTTCGCCTCTAC 3′
	R: 5′ CGTAGTTCTCGTTCCAGTCGT 3′
Hs_TRPV1	F: 5′ GACTTCAAG GCTGTCTTCATCATCC
	R: 3′ CAGGGAGA AGCTCAGGGTGCCC
Hs_ACTB	F: 5′ CCCTGGACTTCGAGCAAGAG 3′
	R: 5′ ACTCCATGCCCAGGAAGGAA 3′
Hs_GEPR1	F: 5′ AGTCGGATGTGAGGTTCAG 3′
	R: 5′ TCTGTGTGAGGAGTGCAAG 3′

F = forward; R = reverse.

## Data Availability

All data will be freely available upon request.

## Supplementary Materials

The following supporting information can be downloaded at: https://www.mdpi.com/article/10.3390/nu14132703/s1, Figure S1: α- and -ENaC positive HBO cells. Co-localization of α-ENaC (**A**) and -ENaC (**B**) antibodies in PLCβ2-positive HBO cells. Left panels transmitted images, blue DAPI- stained cell nuclei, green α-ENaC or -ENaC antibody binding, red PLCβ2 antibody binding. Scale bars = 50 µm; Figure S2: TRPV1 mRNA sequence. TRPV1 mRNA RT-PCR product; Figure S3: Western blots for GPER1, TRPV1, and ACE2 in male, female and OVX female mouse gastric smooth muscle (MGSM) and HBO cells lysates; Figure S4: Co-immunoprecipitation (co-IP) studies of GPER1/AT1R, GPER1/TRPV1 and GPER1/ACE2 in mouse gastric smooth muscle (MGSM) and GPER1/ENaC in HBO cell lysates.
