# Supplementary material for: Relationship between ENaC Regulators and SARS-CoV-2 Virus Receptor (ACE2) Expression in Cultured Adult Human Fungiform (HBO) Taste Cells
Source: Nutrients. 2022 Jun 29;14(13):2703. doi: 10.3390/nu14132703 (PMC9268489; doi:10.3390/nu14132703)
Supplement: Supplementary file 1 [file nutrients-14-02703-s001.zip › nutrients-1766986-supplementary.pdf]

**A**

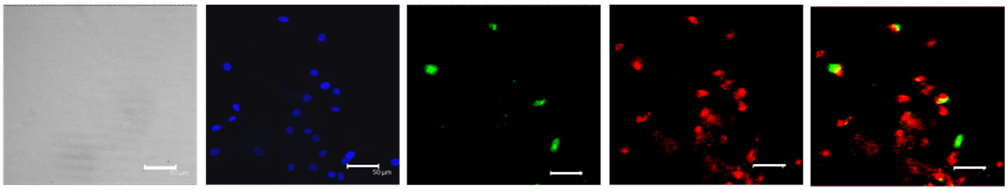

**Rabbit anti-PLCβ2 (1:500, red + goat anti α-ENaC (1:100, green)**

**B**

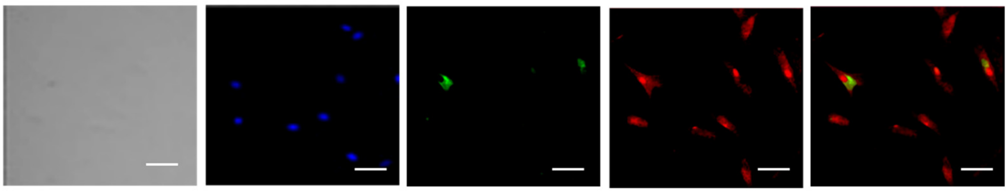

**Rabbit anti-PLCβ2 (1:500, red + goat anti γ-ENaC (1:100, green)**

**Supplementary Figure S1. *α*- and γ-ENaC positive HBO cells.** Co-localization of *α*-ENaC (**A**) and γ-ENaC (**B**) antibodies in PLCβ2-positive HBO cells. Left panels transmitted images, blue DAPI- stained cell nuclei, green *α*-ENaC or γ-ENaC antibody binding, red PLCβ2 antibody binding. Scale bars = 50 μm.

**Range 1: 2225-2535**

| Score | Expect | Identities | Gaps | Strand |
|---|---|---|---|---|
| 551 bits(298) | 2e-160 | 305/311(98%) | 0/311(0%) | Plus/Plus |

```
Query  28    TGCTCAACATGCTCATNNCCCTCATGGGTGAGACTGTCAACAAGATCGCACAGGAGAGCA  87
             ||||||||||||||||  ||||||||||||||||||||||||||||||||||||||||||
Sbjct  2225  TGCTCAACATGCTCATCGCCCTCATGGGTGAGACTGTCAACAAGATCGCACAGGAGAGCA  2284

Query  88    AGAACATCTGGAAGCTGCAGAGAGCCATCACCATCCTGGACACGGAGAAGAGCTTCCTTA  147
             ||||||||||||||||||||||||||||||||||||||||||||||||||||||||||||
Sbjct  2285  AGAACATCTGGAAGCTGCAGAGAGCCATCACCATCCTGGACACGGAGAAGAGCTTCCTTA  2344

Query  148   AGTGCATGAGGAAAGCCTTCCGCTCAGGCAAGCTGCTGCAGGTGGGGTACACACCTGATG  207
             ||||||||||||| ||||||||||||||||||||||||||||||||||||||||||||||
Sbjct  2345  AGTGCATGAGGAAGGCCTTCCGCTCAGGCAAGCTGCTGCAGGTGGGGTACACACCTGATG  2404

Query  208   GCAAGGACGACTACCGGTGGTGCTTCAGGGTGGACGAGGTGAACTGGACCACCTGGAACA  267
             ||||||||||||||||||||||||||||||||||||||||||||||||||||||||||||
Sbjct  2405  GCAAGGACGACTACCGGTGGTGCTTCAGGGTGGACGAGGTGAACTGGACCACCTGGAACA  2464

Query  268   CCAACGTGGGCATCATCAACGAAGACCCGGGCAACTGTGAGGGCGTCAAGCGCACCCTNN  327
             ||||||||||||||||||||||||||||||||||||||||||||||||||||||||||
Sbjct  2465  CCAACGTGGGCATCATCAACGAAGACCCGGGCAACTGTGAGGGCGTCAAGCGCACCCTGA  2524

Query  328   NCTTCTCCCTG  338
              ||||||||||
Sbjct  2525  GCTTCTCCCTG  2535
```

**Supplementary Figure S2. TRPV1 mRNA sequence.** TRPV1 mRNA RT-PCR product, was sequenced, and found to be specific for the TRPV1 gene NM_080705.4

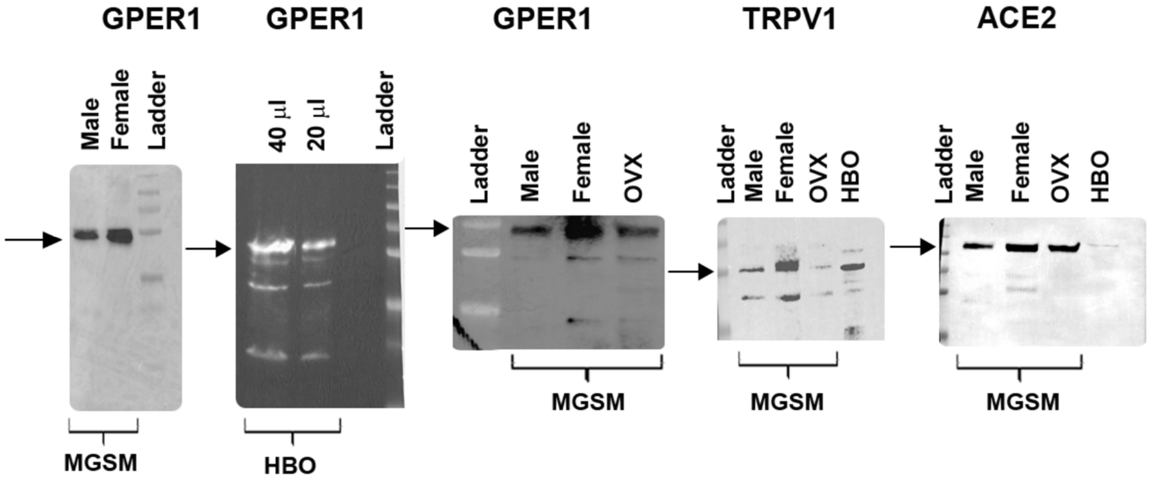


**Supplementary Figure S3. Western blots for GPER1, TRPV1, and ACE2 in male, female and OVX female mouse gastric smooth muscle (MGSM) and HBO cells lysates.** Relative to MGSM from female mice, GPER1 protein expression was lower in males and further decreased in OVX females. Using male, female and OVX females provides a control and a test for GPER1 antibody used in these experiments. Molecular weight (MW) protein ladder bands correspond to 10-250 KDa (bottom to top).

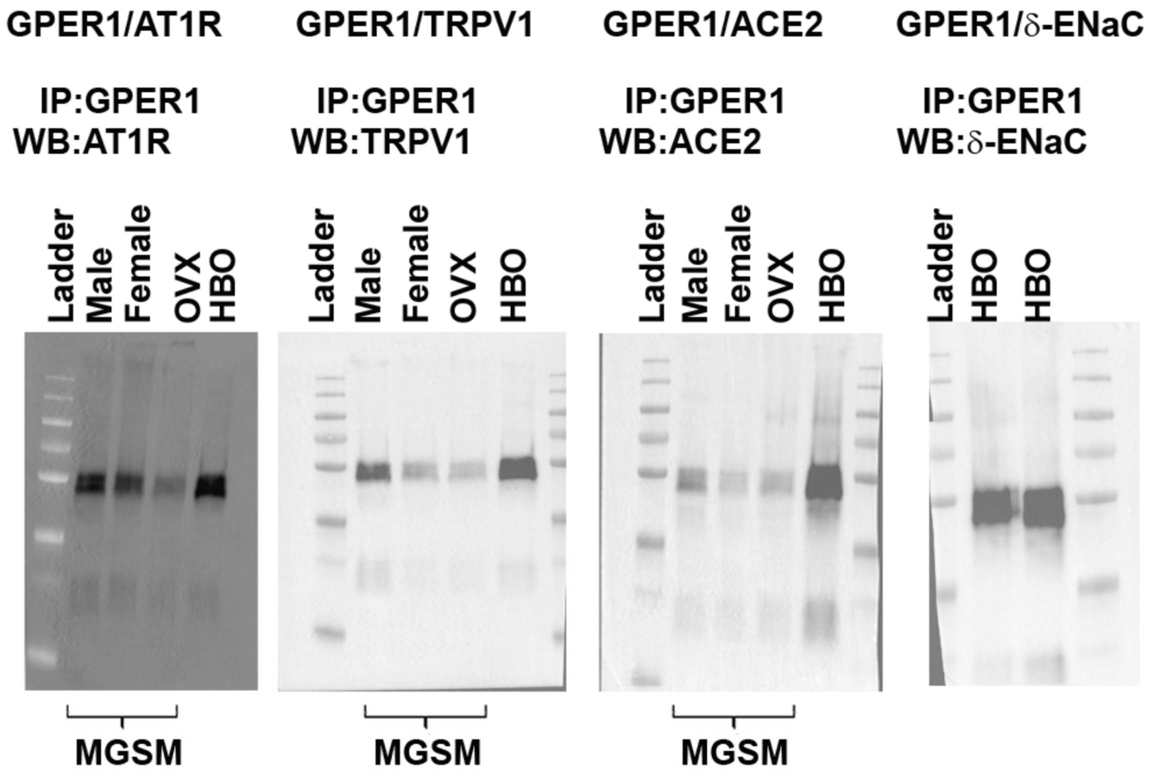


**Supplementary Figure S4. Co-immunoprecipitation (co-IP) studies of GPER1/AT$_1$R, GPER1/TRPV1 and GPER1/ACE$_2$ in mouse gastric smooth muscle (MGSM) and GPER1/δENaC in HBO cell lysates.** GPER1 antibody pulled down AT1R, TRPV1, ACE2 and δ-ENaC in HBO cell lysate. Molecular weight (MW) protein ladder bands correspond to 10-250 KDa (bottom to top).
